# Ranking analysis of *F*-statistics for microarray data

**DOI:** 10.1186/1471-2105-9-142

**Published:** 2008-03-06

**Authors:** Yuan-De Tan, Myriam Fornage, Hongyan Xu

**Affiliations:** 1 College of Life Sciences, Hunan Normal University, Changsha, 410081, China; 2 Institute of Molecular Medicine, the University of Texas-Houston, Houston, Texas 77030, USA; 3 Department of Biostatistics, Medical College of Georgia, Augusta, Georgia, 30912, USA

## Abstract

**Background:**

Microarray technology provides an efficient means for globally exploring physiological processes governed by the coordinated expression of multiple genes. However, identification of genes differentially expressed in microarray experiments is challenging because of their potentially high type I error rate. Methods for large-scale statistical analyses have been developed but most of them are applicable to two-sample or two-condition data.

**Results:**

We developed a large-scale multiple-group *F*-test based method, named ranking analysis of *F*-statistics (RAF), which is an extension of ranking analysis of microarray data (RAM) for two-sample t-test. In this method, we proposed a novel random splitting approach to generate the null distribution instead of using permutation, which may not be appropriate for microarray data. We also implemented a two-simulation strategy to estimate the false discovery rate. Simulation results suggested that it has higher efficiency in finding differentially expressed genes among multiple classes at a lower false discovery rate than some commonly used methods. By applying our method to the experimental data, we found 107 genes having significantly differential expressions among 4 treatments at <0.7% FDR, of which 31 belong to the expressed sequence tags (ESTs), 76 are unique genes who have known functions in the brain or central nervous system and belong to six major functional groups.

**Conclusion:**

Our method is suitable to identify differentially expressed genes among multiple groups, in particular, when sample size is small.

## Background

Microarray gene expression technology, which profiles the expression of multiple genes in parallel [1,2], affords the means for globally exploring physiological and pathological processes [3] regulated by the coordinated expression of thousands of genes [4]. However, identification of genes that are differentially expressed in large-scale gene expression experiments requires global statistical methods rather than traditional statistical methods based on single hypothesis testing. A variety of multiple-testing procedures, such as the Bonferroni procedure, Holm procedure [5], Hochberg procedure [6], Benjamini-Hochberg (BH) procedure [7], and Benjamini-Liu (BL) procedures [8] have already been developed for testing a large family of null hypotheses. The first three methods bound the family-wise-error rate (FWER) that is the probability of at least one false positive over all tests and hence remain too stringent and have lower power for finding genes from the real data sets. The last two methods have an upper bound for the false discovery rate (FDR) with both strong and weak controls [9] and require a large sample size for valid p-values. Tusher et al. [9] has proposed a ranking statistic approach based on permutation for resampling. However, permutation is not a desirable approach to estimating null distribution in microarray data [10-12] because in general a microarray dataset has a large number of genes but small sample sizes [13] due to cost. Permutation fails to remove treatment effect and due to small sample sizes the difference of treatment effects between permutated groups may become a main component in differences between group means so that the estimated null distribution is not well approximate to the true null distribution ([13] and also see Appendix in Tan et al. [14]). For example, Xie et al. [12] found that the estimated null F-distribution based on permutation has a larger variance and a heavier tail compared to the true null F-distribution, which leads to a potential loss of power. Similar phenomenon was also observed in comparison of the estimated null t-distribution to the true null t-distribution [14]. To remove the group or treatment effects on the estimated null distribution, Tan et al. [14] developed a random splitting (RS) approach. Since treatment effects are completely eliminated, the estimated null distribution obtained by the RS method is smooth, stable and approximate true null distribution well.

For the multi-class microarray data, the analysis of variance (ANOVA) is useful to identify differentially expressed genes [4]. In ANOVA, the *F*-test is a generalization of the *t*-test that allows for comparison of more than two samples [15]. However, due to small sample sizes, the classical F-test is also subject to the same problems as the *t*-test: bias and unstable estimates of gene-specific variances. To tackle this issue, many authors [15-19] proposed modified *F*-statistics. However, like the classical *F*-test, these modified *F*-tests still suffer from high false-positive rates because (i) the sample size is often so limited that the asymptotic F distribution is not accurate enough to obtain a valid *p*-value and (ii) they appeal to multiple-testing procedures such as the Bonferroni procedure or the BH-procedure. As mentioned above, these multiple-testing procedures have a basic requirement that sample sizes are large enough for valid p-values. In microarray data, the requirement is not realistic. Based on consideration of these problems, we here propose a novel statistical method for the analysis of multi-class gene-expression data called Ranking Analysis of *F*-statistics (RAF). RAF is a natural extension of our previous work, i.e., the ranking analysis of microarrary (RAM) for two-class *t*-tests [14]. It works on finding genes that are differentially expressed among multiple treatment groups by comparing the ordered real *F*-statistics with the ordered estimated null *F*-statistics and implementing a two-simulation strategy to estimate the false discovery rate (FDR).

## Methods

### Animal model and design

Studies were performed on male stroke-resistant SHR/N (CRiv) (SHRSR) and stroke-prone SHR/A3 (Heid) (SHRSP) rats from a breeding colony maintained by the investigators as previously described [20]. Age-matched male rats from each strain (N = 12 SHRSP and 12 SHRSR) were fed a standard rat chow and water ad libitum until age 8 weeks. Subsequently, animals from each strain were randomized to one of 2 dietary regimens (N = 6 in each strain × diet group): a "stroke-permissive diet" high in sodium (HS) (0.63% potassium, 0.37% sodium) and 1% NaCl drinking solution; a "stroke-protective diet" low in sodium and high in potassium (LS) (1.3% potassium, 0.35% sodium) and regular drinking water. All animals were housed at 23°*C *on a 12-hour light-dark cycle. Rats were sacrificed at 12 weeks of age, and brain tissue was collected for RNA extraction and subsequent microarray analysis. The study protocols were approved by the Animal Care Committee of the University of Texas – Houston. Since strain and dietary factor each have only two levels, we here treated them as one-way in statistical analysis instead of two-way, that is, we are neither interested in strain effects alone nor in dietary effects alone but focus on their combined contributions to gene expression. Thus, HS-SHRSPs, LS-SHRSPs, HS-SHRSRs, and LS-SHRSRs are viewed as four treatment groups for the purpose of the analyses.

### Microarray experiment

Microarray analysis was performed as described by Lockhart et al. [21]. Briefly, 10 *μ*g total RNA extracted from each of the 24 rats was used to synthesize cDNA, which was then used as a template to generate biotinylated cRNA. cRNA was fragmented and hybridized to a Test2 chip to verify quality and quantity of the samples. Each sample was then hybridized to a RGU34A array (Affymetrix, Santa Clara, CA). After hybridization, each array was washed and scanned, and fluorescence values were measured and normalized using the Affymetrix Microarray Suite v. 5.0 software.

### Ranking *F*-Test

Let *x*_*gij *_be the expression value for replicate *j *of gene *g *in group *i *where *g *= 1,..., *N *(number of genes), *j *= 1,..., *r*_*gi *_(number of replicate observed values of gene *g *in group *i*) and *i *= 1,..., *n *(number of groups). The traditional *F*-statistic in one-way ANOVA may be expressed as

(1) $$F_{g}=\frac{\sigma^{2}(G_{g})}{\sigma^{2}(e_{g})}$$

where *σ*^2 ^(*G*_*g*_) and *σ*^2 ^(*e*_*g*_) are inter- and intra-group variances of the expression values of gene *g*, respectively. In the conventional *F*-tests, for example, significance of *p *= 0.01 in the context of the standard *F *distribution is for a single hypothesis to be tested; therefore, it is unsuitable to microarray data because in a microarray experiment for 10,000 genes we would expect to identify 100 genes by chance [9]. To address this problem, an alternative approach is to rank genes by magnitude of their *F *values so that *F*_1 _is the largest value, *F*_2 _is the second largest value, etc., and *F*_g* _is the g*th largest value where g* is a rank position of gene *g*. Thus, we have a ranking *F*-test where

(2) *F*_g* _- *f*_g* _> Δ

indicates that the expression variation of gene *g *among multiple groups (or multiple conditions) is significant. In Eq. (2), *f*_g* _is the expectation of *F*_g* _under the null hypothesis and Δ is a given threshold.

### Estimation of *f*_g*_

In the ANOVA framework, we have

(3) $$\begin{array}{c} \sigma^{2}(G_{g})=\frac{\sum_{i=1}^{n}r_{gi}{\left({\bar{x}}_{gi}-{\bar{x}}_{g}\right)}^{2}}{n-1}=\frac{\sum_{i=1}^{n}r_{gi}{\left[(\tau_{gi}+{\bar{e}}_{gi})-(\mu_{g}+{\bar{e}}_{g})\right]}^{2}}{n-1}\\ =\frac{\sum_{i=1}^{n}r_{gi}(\tau_{gi}-\mu_{g})+\sum_{i=1}^{n}r_{gi}{\left({\bar{e}}_{gi}-{\bar{e}}_{g}\right)}^{2}}{n-1}\\ =\sigma^{2}(\tau_{g})+\sigma^{2}({\bar{e}}_{g}) \end{array}$$

where ${\bar{x}}_{gi}=\tau_{gi}+{\bar{e}}_{gi}$ and ${\bar{x}}_{g}=\mu_{gi}+{\bar{e}}_{g}$; *τ*_*gi *_and ${\bar{e}}_{gi}$ are treatment effect and average random error in group *i*, respectively; ${\bar{x}}_{g}$ and ${\bar{e}}_{g}$ are the overall observed mean and the overall average error for gene *g*, respectively, and *μ*_*g *_is the overall expected mean for gene *g *and *r*_*gi *_is the number of replicate observed values of gene *g *in group *i*. Therefore, the inter-group variance *σ*^2 ^(*G*_*g*_) consists of two parts: variance of treatment effects on expression of gene *g*, *σ*^2 ^(*τ*_*g*_), and average error variance, *σ*^2 ^(${\bar{e}}_{g}$). Thus, *F*-statistic can be rewritten as

(4) $$F_{g}=\frac{\sigma^{2}(G_{g})}{\sigma^{2}(e_{g})}=\frac{\sigma^{2}(\tau_{g})+\sigma^{2}({\bar{e}}_{g})}{\sigma^{2}(e_{g})}=\frac{\sigma^{2}(\tau_{g})}{\sigma^{2}(e_{g})}+\frac{\sigma^{2}({\bar{e}}_{g})}{\sigma^{2}(e_{g})}=\frac{\sigma^{2}(\tau_{g})}{\sigma^{2}(e_{g})}+f_{g}.$$

Therefore, the null hypothesis is equivalent to *F*_*g *_= *f*_*g *_because *σ*^2 ^(*τ*_*g*_) = 0 under the null hypothesis. Note that *σ*^2 ^(*τ*_*g*_) = 0 means the treatment effects *τ*_*gi *_= ... = *τ*_*gn *_= *μ*_*g*_. In order to do a ranking *F*-test, it is necessary to obtain a good estimate of *f*_g* _. In the two-group scenario, Tusher et al. [9] employed a permutation approach to estimate the expected *t*-statistics. The permutation process cannot completely clear the treatment effect in the ranked *d*-statistics so that the estimated ranked *d*-statistics distribution is biased against its null distribution and unstable, in particular, when sample sizes are small (see Appendix A in Tan et al., [14]). Tan et al. [14] developed a "Randomly Splitting" (RS) approach to estimate the null distribution of *t*-statistics. In this study, we extended the RS approach to estimating the null distribution of *F*-statistics.

In the RS approach, one sample consisting of *r*_*gi *_replicates is drawn from group *i*. Since only one sample is drawn from a group, sample *i *represents group *i*. Within a sample all the observed expression values of gene *g *come from the same group. These values have the same overall expected mean *μ*_*g *_and the same treatment effect *τ*_*gi *_on expression of gene *g *except for expression noises. A sample of *r*_*gi *_replicate values for gene *g *is randomly split into two sub-samples denoted by s = 1 and s = 2. If let ${\bar{x}}_{gis}^{J}$ be the mean of sub-sample *s *of sample *i *for gene *g *at split *J*(*J *= 1,..., M), then ${\bar{x}}_{gis}^{J}$ can be expressed as

(5) $${\bar{X}}_{gis}^{J}=\mu_{g}+\tau_{gi}+{\bar{e}}_{gis}^{J}$$

where ${\bar{e}}_{gis}^{J}=\sum_{j=1}^{m_{gis}^{J}}e_{gisj}^{J}/m_{gis}^{J}$ and $m_{gis}^{J}$ and $e_{gisj}^{J}$ are replicate number and noise in the observed expression value *j *in sub-sample *s *in group *i *for gene *g *at split *J*, respectively. ${\bar{e}}_{gi}$ is estimated by the difference between two sub-sample means in sample *i *for gene *g *at split *J*,

(6) $$\begin{array}{l} {\bar{e}}_{gi}^{J}=\frac{1}{2}\left({\bar{x}}_{gi1}^{J}-{\bar{x}}_{gi2}^{J}\right)\\ =\frac{1}{2}\left[\left(\mu_{g}+\tau_{g}+{\bar{e}}_{gi1}^{J}\right)-\left(\mu_{g}+\tau_{g}+{\bar{e}}_{gi2}^{J}\right)\right].\\ =\frac{1}{2}\left({\bar{e}}_{gi1}^{J}-{\bar{e}}_{gi2}^{J}\right) \end{array}$$

It can be seen from Eq. (6) that *μ*_*g *_and *τ*_*gi *_are cleared in difference between two sub-sample means, which is unrelated to sample size. Thus, the average random error variance *σ*^2 ^(${\bar{e}}_{g}$) in Eq.s (3) and (4) can be estimated by

(7) $$\sigma^{2}({\bar{e}}_{g}^{J})=\frac{\sum_{i=1}^{n}r_{gi}{\left({\bar{e}}_{gi}^{J}-{\bar{e}}_{g}^{J}\right)}^{2}}{n-1}$$

where ${\bar{e}}_{g}^{J}=\sum_{i=1}^{n}{\bar{e}}_{gi}^{J}/n$ is estimate of mean (${\bar{e}}_{g}$) of expression noise of gene *g *among groups at split J. Variance *σ*^2^(${\bar{e}}_{g}$) is an estimate of expectation (*σ*^2^(graphic file="1471-2105-9-142-i17.gif"/>)) of inter-group variance (*σ*^2 ^(*G*_*g*_)) under the null hypothesis at split J. We therefore have

(8) $$f_{g}^{J}=\frac{\sigma^{2}({\bar{e}}_{g}^{J})}{\sigma^{2}(e_{g})}.$$

Note that since treatment effect is completely removed from the difference between two sub-sample means, the difference is pure noise. We rank $f_{g}^{J}$ across all *g *and let $f_{g*}^{J}$ denote the value in ordered position g* at split *J*. After running M splits, we have M values of $f_{g*}^{J}$ for position g*. Thus *f*_g* _in Eq. (2) can be estimated by the average of $f_{g*}^{J}$ over all *M *splits, i.e., ${\bar{f}}_{g*}=\sum_{J=1}^{M}f_{g*}^{J}/M$.

### Estimation of FDR

To identify genes whose expression is significantly changed among multiple conditions, it is necessary to estimate the FDR for a given threshold [7,22]. Here we propose a two-simulation approach for FDR estimation [14]. Consider a series of threshold values Δ_*k*_(*k *= 1,...*L*) and let *N*_*k *_be the number of genes that are claimed as significant by RAF at threshold Δ_*k*_. *N*_*k *_comprises two parts: the number *N*_*k*_(*t*) of the true positives and the number *N*_*k*_(*f*) of the false positives, i.e., *N*_*k *_= *N*_*k*_(*t*) + *N*_*k*_(*f*). Thus, given a threshold Δ_*k*_, FDR is defined as *λ*_*k *_= *N*_*k*_(*f*)/*N*_*k*_. *N*_*k*_(*f*) is unknown, hence *λ*_*k *_must be estimated. Many approaches such as BH procedure [7,22], BL procedure [8], Storey's procedure [23,24], and Pounds and Cheng's procedure [25] have been proposed to estimate the FDR. These approaches, however, are based on the assumption that the tests are independent. As mentioned previously, this assumption may not be met in practice. Therefore, these methods may not be suitable to our ranking test. Based on the fact that sampling distribution fluctuates around the expected distribution via permutation, Tusher et al. [9] developed a permutation-based estimator to estimate FDR in the ranking tests. It has been proved, however, in theory and in simulation that when the sample sizes are small, the number of permutations is very limited so that the treatment effects cannot be removed in the permutated data [14]. As a result, the estimator is biased for a given threshold. Here we extend the interval approach by Tan et al. [14] to the ranking analysis of F-statistics. In this approach, we first construct an estimated interval of the true FDR, and then we find a reasonable estimate of FDR. This interval is based on the complete and partial null distributions given by two simulations.

In simulation 1, for each gene, *n *samples (groups) each having *r *replicates are generated from normal distributions with a set of sample means (${\bar{y}}_{g1},...,{\bar{y}}_{gn}$) and a set of sample error variances [*s*^2 ^(*e*_*g*1_),..., *s*^2 ^(*e*_*gn*_)]. Here we set ${\bar{y}}_{g1}=....={\bar{y}}_{gn}={\bar{x}}_{gi}$ and *i *is a randomly chosen group from the observed data, for each of a half of the genes with the null effect that the group variance is zero, i.e., *σ*^2 ^(*G*_*g*_) = 0 and ${\bar{y}}_{gi}={\bar{x}}_{gi}$ for each of the other half with unknown effect that the group variance is larger than or equal to zero, i.e., *σ*^2 ^(*G*_*g*_) ≥ 0. *s*^2 ^(*e*_*gi*_) is set to be equal to *σ*^2 ^(*e*_*gi*_) where ${\bar{x}}_{gi}$ and *σ*^2 ^(*e*_*gi*_) are the observed values from the real microarray data set.

**Figure 1:**
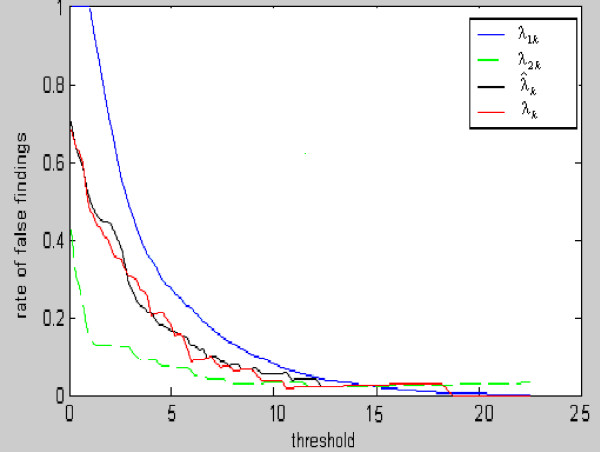
**Profile of estimates of FDRs for a series of thresholds**. *λ*_1*k *_and *λ*_2*k *_are two threshold functions from simulations 1 and 2 and were used to construct an estimation interval for estimate of FDR at threshold Δ_*k*_. *λ*_*k *_and ${\hat{\lambda }}_{\text{k}}$ are true and estimated FDRs at threshold Δ_*k*_, respectively, where *k *= 1, 2,...,*L*.

*B *sets of simulation data are obtained from this procedure. Each is subject to the ranking analysis described in the previous section. For simulation data set *b*, every ranked position has thus its corresponding *F *value that is denoted by $F_{g*1}^{L}$(*b *= 1,..., *B*). Here those that are called significant by comparing $F_{g*1}^{b}$ to ${\bar{f}}_{g*}$ at a given threshold Δ_*k *_are counted as $N_{1k}^{b}$ across all ranking positions. Let $N_{1k}=\max_{J=1}^{B}N_{1k}^{b}$ Given the fact that a small part of genes have unequal means in the samples, the simulation data set produces a partially null F-distribution. In other words, it may produce $N_{1k}^{b}>N_{k}(f)$, possibly leading to *λ*_*k *_= *N*_1*k*_/*N*_*k *_> 1. To avoid this situation, suppose *N*_1*k*_(*k *= 1,..., *L*) takes the maximum value *N*(*m*) at Δ_*k *= *m*_, we define

(9) $$\lambda_{1k}=\frac{2N_{1k}}{N(m)+N_{1k}}$$

as a function of threshold Δ_*k *_for estimating FDR where we set *N*_1*k *_= *N*(*m*) for all Δ_*k *_< Δ_*m*_(*k *= 1,..., *L*). Obviously, *λ*_1*k *_is a decreasing function of the threshold and bounded between 0 and 1. For example, *λ*_1*k *_= 1 when *N*_1*k *_= *N*(*m*), and *λ*_1*k *_= 2/3 when *N*_1*k *_= *N*(*m*)/2. Results from the simulation study in Figure 1 indicate that *λ*_1*k *_≥ *λ*_*k *_(true value of FDR at threshold Δ_*k*_) when threshold Δ_*k *_is smaller than a value Δ* but *λ*_1*k *_≤ *λ*_*k *_when Δ_*k *_> Δ* (see Figure 1).

The second simulation for estimating FDR is carried out in the following fashion. *n *samples (groups) each having *r *replicates for each gene are generated from normal distributions with a set of sample means, ${\bar{y}}_{g1}=....={\bar{y}}_{gn}={\bar{x}}_{gi}$ and a set of sample variances *s*^2 ^(*e*_*g*1_) = *σ*^2 ^(*e*_*g*1_),..., *s*^2 ^(*e*_*gn*_) = *σ*^2 ^(*e*_*gn*_).

We also produce *B *sets of data from simulation 2. As in simulation 1, for each simulation data set, every ranked position also has its corresponding *F*-value denoted as $F_{g*2}^{b}$ (*b *= 1,..., B). Let $F_{g*2}=\min_{b=1}^{B}F_{g*2}^{b}$. The positives found by comparing $F_{g*2}^{b}$ to *F*_g*2 _at a given threshold Δ_*k *_are counted as $N_{2k}^{b}$ across all ranking positions. Here let $N_{2k}=\sum_{b=1}^{B}N_{2k}^{b}/B$. Unlike the first simulation, here the simulation data sets produce *B *null *F*-distributions, so *N*_2*k *_should be approximate to the true number of false positives *N*_*k*_(*f*). However, when threshold Δ_*k *_is large, it is possible to have *N*_*k *_= 0 so that *N*_2*k*_/*N*_*k *_is undefined. To avoid this situation, we define

(10) $$\lambda_{2k}=\frac{N_{2k}}{N_{k}+N_{2k}}$$

as the second function of threshold (see Figure 1). In particular, we let *λ*_2*k *_= 1 if *N*_*k *_= *N*_2*k *_= 0 because *λ*_2*k *_= 1 when *N*_*k *_= 0 and *N*_2*k *_> 0.

Thus, an interval for FDR estimation at threshold Δ_*k *_can be constructed between *λ*_1*k *_and *λ*_2*k *_. The third function of threshold for FDR estimation is given as

*                         λ*_3*k *_= *α*_*k*_*λ*_1*k *_+ *β*_*k*_*λ*_2*k*_

where *α*_*k *_= min(*λ*_1*k*_, *λ*_2*k*_)/(*λ*_1*k *_+ *λ*_2*k*_) and *β*_*k *_= 1 - *a*_*k*_. *λ*_3*k *_plays the role of weight in balancing *λ*_1*k *_and *λ*_2*k *_. Therefore, at threshold Δ_*k*_, a putative probability that a false discovery is found in the genes called significant by RAF is

(12) $${\hat{\lambda }}_{k}=\frac{1}{3}(\lambda_{1k}+\lambda_{2k}+\lambda_{3k}).$$

Note that as shown in the simulation result section, *λ*_2*k *_is an underestimate of *λ*_*k *_and *λ*_1*k *_is an overestimate of FDR when the threshold Δ_*k *_< Δ*. However, the situation is reversed when threshold Δ_*k *_> Δ*. This is because *N*_1*k *_becomes very small when Δ_*k *_> Δ* so that *λ*_1*k *_becomes very small whereas, from Eq. (10), *λ*_2*k *_slowly decreases if *N*_*k *_> *N*_2*k *_or increases if *N*_*k *_<*N*_2*k *_as threshold increases. In addition, when the microarray data have no treatment effects for all the genes detected, then *λ*_1*k *_= *λ*_2*k *_= *λ*_3*k *_= 1, leading to ${\hat{\lambda }}_{k}$ = 1

In order to smooth ${\hat{\lambda }}_{k}$ between thresholds Δ_*k *_and Δ_*k*+1_, we define a recursive formula modifying the probability ${\hat{\lambda }}_{k}$ as

(13) $${\hat{\lambda }}_{k}=\lambda_{k}p_{k}+\lambda_{k+1}q_{k}$$

where *p*_*k *_= (*N*_*k *_- *N*_*k*+1_)/(1 + *N*_*k *_- *N*_*k*+1_) and *q*_*k *_= 1 - *p*_*k*_. Eq. (13) suggests that *λ*_*k*+1 _= *λ*_*k *_if *N*_*k *_= *N*_*k*+1_. The number of false discoveries among those found to be significant at threshold Δ_*k *_in the observed data is estimated by ${\hat{N}}_{k}(f)={\hat{\lambda }}_{k}N_{k}$. Figure 1 shows that the curve of ${\hat{\lambda }}_{k}$ agrees well with that of *λ*_*k*_.

## Results

### Estimation of the null distribution of *F*-statistics

To examine if the empirical distributions obtained by the RS approach are appropriate for the analysis of the expression data, we simulated a microarray data set consisting of 3770 genes and four groups each having 6 replicates using one group mean and error variance for each gene. Thus, the simulation without treatment effect generated a set of pure noise data.

**Figure 2:**
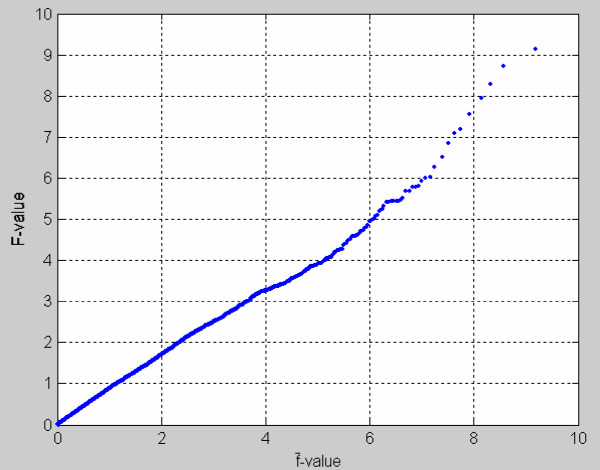
**The dot-plot of *F*-values versus $\bar{f}$-values**. *F*-values and $\bar{f}$-values obtained from the simulated microarray data of 3770 genes were ranked where $\bar{f}$-values were yielded by the random splitting approach. All ranked *F *- $\bar{f}$ dots roughly fall on a diagonal line as expected by two sets of the same ranked distributions.

A set of 3770 *F*_*k *_values was computed from the simulated data set. We applied our RS approach to this simulated data set to generate ${\bar{f}}_{k}$ over 50 splits. This set of 3770 *F*_*k *_values formed null distribution of *F*-statistics, which is called *f*-distribution. To display the profile that our estimate of *f*-distribution is approximate to the null *f*-distribution, we plotted the ranked *F*_*k *_versus ranked ${\bar{f}}_{k}$. The result displays in Figure 2 where all ranked *F *- $\bar{f}$ dots roughly fall on a diagonal line as expected by two sets of the same ranked distributions. These results suggest that the $\bar{f}$-distribution is indeed an approximate estimate of *f-*distribution.

### Estimation of FDR

Since it is in general unknown if a given gene expresses differently among multiple conditions, it is not necessarily best to use real data of gene expression to evaluate an FDR estimator. But simulation is a useful approach to doing such a task. Therefore, we also conducted a computer simulation for comparing expression status (significant or not) of a gene identified by a method with its real status. This simulation was also based on our real data set of 3770 genes. Treatment effect *τ *on expression variation was set for 30 % of the genes and assigned in 4 groups. The mean expression value of gene *g *was set ${\bar{y}}_{g1}={\bar{x}}_{g}+2\tau ,{\bar{y}}_{g2}={\bar{x}}_{g}+\tau ,{\bar{y}}_{g3}={\bar{x}}_{g}-\tau$ and ${\bar{y}}_{g4}={\bar{x}}_{g}-2\tau$ for the 4 groups where *τ *= 100*U*, 0 <*U *≤ 1, ${\bar{x}}_{g}$ is overall observed average for gene *g*, and each group has 6 replicates. Obviously, treatment effect *τ *on expression changes randomly with genes in our simulation, which would make it more difficult to identify differentially expressed genes than the simulations with a fixed treatment effect. Figure 1 displays a comparison between RAF estimated and true FDRs. One can see that the RAF estimate curve is very close to the true FDR curve given a series of thresholds.

### Efficiencies of different methods in finding genes differentially expressed among multiple groups

To evaluate different methods, we generated 30 simulation data sets of 3770 genes with the same simulation procedure described above where treatment effect *τ *was randomly assigned to 10% of the genes in 4 groups, each with 6 replicates. We compared four typical methods with these simulation datasets, of which the Bonferroni (B) procedure and Benjamini-Hochberg (BH) procedure are conventional multiple-testing procedures based on a series of *p*-values obtained from the classical *F*-test; SAM is a ranking method using the Fisher linear discrimination [9]. Our method also is a ranking method but based on the classical *F*-test. Although the *F*-test based on the hierarchical error model (HEM) proposed by Cho and Lee [26] also is suitable to multiple-sample data, the HEM method has consistent performance with the SAM and has no estimate of FDR. Therefore we did not take the HEM method into account of our comparisons among methods. Table 1 summarizes the results obtained by applying these four methods to the 30 simulation datasets where efficiency of a method in finding genes differentially expressed among multiple groups was comprehensively evaluated by averaging number of called significances (NCS), estimated number of false positives (ENFP), true number of false positives (TNFP), and differences (*d *values) between ENFPs and TNFPs within a given range of FDR over these 30 simulation data sets. Here we measure the conservativeness of a method by the conservative degree *C*(*d *≥ 0), defined as the proportion of simulations with *d *≥ 0. In Table 1, as expected, the B procedure gave the most conservative findings and the lowest power among these methods. Similarly, the BH procedure also yielded a very conservative result in which 96.7 percent of ENFPs were larger than TNFPs, in other words, conservation degree reached 96.7%. For the two ranking methods, Table 1 displays the results in the cases of FDR at 6 levels 0.04 <*λ *≤ 0.05, 0.03 <*λ *≤ 0.04, 0.02 <*λ *≤ 0.03, 0.01 <*λ *≤ 0.02, 10^-4 ^<*λ *≤ 0.01, and *λ *< 10^-4 ^. It is clear that RAF has slightly larger means of NCS at all these 6 FDR levels than SAM. In RAF, the means of ENFP are all higher than the means of TNFP while in SAM the means of ENFP are all less than the means of TNFP. Table 1 also shows that RAF has 75~86.2% conservation degree in estimates of false positives under 5% of FDR whereas SAM has 23~66% of conservation degrees. These results suggest that RAF has the highest efficiency in finding genes differentially expressed among these four methods.

**Table 1:** Efficiencies of different methods in identifying genes differentially expressed among four groups each with 6 replicates in 30 simulated datasets

		NGCS			ENFP			TNFP			Difference between ENFP and TNFP		
		
Method	FDR	Mean (SD)	Min	Max	Mean (SD)	Min	Max	Mean (SD)	Min	Max	$\left|\bar{d}\right|$	*Var *(*d*)	*C*(*d *≥ 0)
B procedure	*λ *= 0.05	59.6 (6.6)	46	73	3.0 (0.3)	2	4	0.0(0.0)	0	0	3.0		100%
													
BH Procedure	*λ *= 0.05	102.2 (9.9)	81	119	4.8 (1.0)	4	6	1.6 (1.4)	0	6	3.2		97%
													
SAM	0.04 <*λ *≤ 0.05	111.5(14.3)	89	129	5.1 (0.6)	5	6	5.6(2.8)	2	12	2.0	6.7	56.5%
	0.03 <*λ *≤ 0.04	106.8(13.2)	84	119	3.7 (0.6)	3	5	3.8(2.3)	0	8	1.5	4.0	66.7%
	0.02 <*λ *≤ 0.03	96.2(12.5)	80	119	2.3 (0.6)	1	3	3.1(1.7)	1	6	1.4	3.1	39.4%
	0.01 <*λ *≤ 0.02	91.0(12.7)	71	107	1.3 (0.47)	1	2	1.6(1.2)	0	4	0.9	1.1	67.5%
	0.00 <*λ *≤ 0.01	98.7(6.6)	94	108	0.9 (0.1)	1	1	1.5(1.1)	0	3	1.0	1.9	36.4%
	*λ *= 0.00	82.9(11.0)	66	108	0.0 (0.0)	0	0	1.0(0.6)	0	3	1.0	1.4	23.1%
													
RAF	0.04 <*λ *≤ 0.05	115.1 (9.2)	96	131	5.1 (0.4)	4	6	4.4(2.7)	1	9	2.2	7.3	75.0%
	0.03 <*λ *≤ 0.04	110.6(12.2)	85	128	3.9 (0.6)	3	5	3.2(2.1)	1	8	1.6	3.9	79.2%
	0.02 <*λ *≤ 0.03	103.6 (10.6)	86	120	2.7 (0.5)	2	3	2.1(1.5)	0	6	1.3	2.8	81.8%
	0.01 <*λ *≤ 0.02	100.7 (10.8)	81	118	1.7 (0.5)	1	2	1.1(0.9)	0	3	0.9	1.3	75.8%
	0.00 <*λ *≤ 0.01	100.8 (4.1)	96	112	1.1 (0.2)	1	2	0.7(1.0)	0	3	0.9	1.4	77.8%
	*λ *= 0.00	83.8 (7.1)	69	95	0.0 (0.0)	0	0	0.1(0.3)	0	1	0.1	0.1	86.2%

FDR, false discovery rate; NGCS, number of genes called significant; ENFP, estimated number of false positives; TNFP, true number of false positives.

$\left|\bar{d}\right|=\frac{1}{N_{\lambda }}\sum_{k=1}^{N_{\lambda }}\left|d_{k}\right|$ where *d*_*k *_= *ENFP*_*K *_- *TNFP*_*K *_and *N*_*λ *_is number of *x *<*λ *≤ *y *in 30 simulations. $C(d\geq 0)=\sum_{k=1}^{N_{\lambda }}{\text{I}}_{k}/N_{\lambda }$ where I_*k *_= 1 if *d*_*k *_≥ 0, otherwise, I_*k *_= 0. $Var(d)=\frac{1}{N_{\lambda }-1}\sum_{k=1}^{N_{\lambda }}{\left(d_{k}-0\right)}^{2}=\frac{1}{N_{\lambda }-1}\sum_{k=1}^{N_{\lambda }}d_{k}^{2}$.

We also generated a simulated data set of 3770 genes where treatment effect *τ *was randomly assigned to 10% of genes but sample size for each group was changed from 6 replicates to 4. Table 2 displays the results obtained by SAM and RAF from this data set. It can be seen that SAM has very high FDRs while RAF still works well and detects 9 genes without false positives.

**Table 2:** Comparison between SAM and RAF in finding genes differentially expressed among four classes in a simulated data set of small sample size (n = 4)

SAM				RAF				
	
Delta	Number of significances	Number of false positives	Estimated FDR	Delta	Number of significances	Number of false positive	Estimated FDR	True FDR
0.037534	10	5.6	0.56	0.01253	16	6	0.375	0.125
0.044668	10	5.6	0.56	0.37608	13	4	0.308	0.077
0.045738	10	5.6	0.56	0.74013	13	3	0.231	0.077
0.050144	9	4.7	0.52	1.10516	12	2	0.167	0.083
0.052423	9	4.7	0.52	1.47167	11	1	0.091	0
0.055937	9	4.7	0.52	1.84017	10	1	0.100	0
0.059564	9	4.7	0.52	2.58527	9	0	0	0
0.060798	9	4.7	0.52					
0.062046	9	4.7	0.52					
0.063305	0	0	0					

### Array findings by RAF

**Figure 3:**
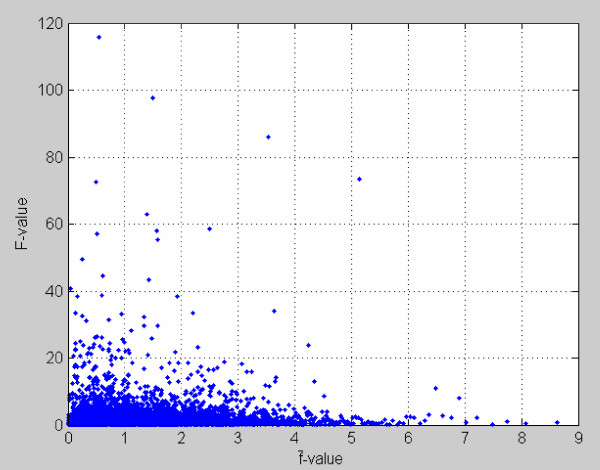
**The scatter plot of *F*-values versus $\bar{f}$-values**. *F*-values were observed from real microarray data set and $\bar{f}$-values yielded by random splitting approach are an estimate of null *f*-distribution.

We obtained a set of the observed data in which expression of 3770 genes was measured among four treatment groups HS-SHRSPs, LS-SHRSPs, HS-SHRSRs, and LS-SHRSRs. This set of microarray data is readily applicable to our ranking *F*-test analysis. Figure 3 shows a scattered-dot plot of *F*-values versus $\bar{f}$-values obtained by the RS approach.

**Figure 4:**
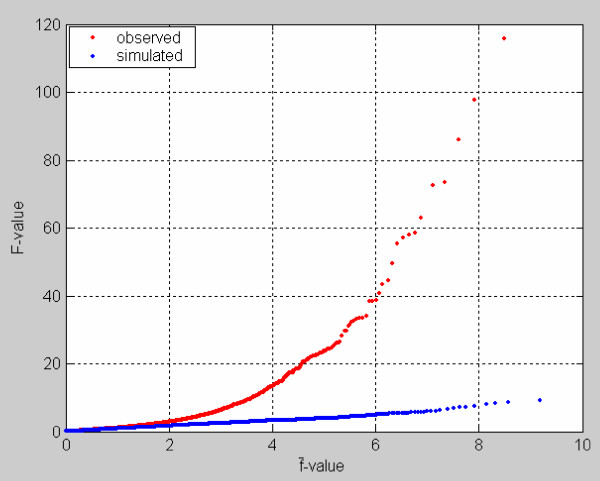
**Comparison between the observed (red) and simulated (blue) plots of *F*-values versus $\bar{f}$-values**. *F*-values were observed from real (red) and simulated (blue) microarray data sets of 3770 genes and 6 replicates. $\bar{f}$-value yielded by randomly splitting approach is an estimate in null *f*-distribution. *F*-distribution from simulated data set without treatment effects is null distribution. Ranked *F*-values corresponds to ranked $\bar{f}$-values.

Figure 4 compares the observed plot of ranked *F *- $\bar{f}$ to the simulated one. One can see from Figure 4 that the observed *F *- $\bar{f}$ plot begins to deviate from the simulated *F *- $\bar{f}$ plot at about $\bar{f}$ = 2.1, suggesting that a part of the F-statistics deviates from the $\bar{f}$-distribution. This result underscores that these genotypes and diet feeds significantly impact on expression of a portion of genes in rat with respect to stroke.

The numbers of genes whose expression is significantly different among the four groups HS-SHRSPs, LS-SHRSPs, HS-SHRs, and LS-SHRs, are found to be 392, 145, and 107 by our RAF under estimates of FDR of 4.8, 0.7, and <7.0% (see Table 3), respectively. These 107 genes with FDR<0.7% are listed in the Additional file 1. Among these 107 identified probes, 31 belong to the expressed sequence tags (ESTs), 76 are unique genes who have known functions in the brain or central nervous system and belong to six major functional classes: (a) neurotransmission such as neurexin III-alpha, Neurodap-1, non-neuronal enolase (NNE), beta isoform of catalytic subunit of cAMP-dependent protein kinase; (b) cell signaling and transportation such as trans-Golgi network integral membrane protein (TGN38), glutamate transporter, alternatively spliced GTP-binding protein alpha subunit intracellular, signal regulatory protein alpha, synaptic vesicle protein 2B (SV2B), L-type amino acid transporter 1 (LAT1), N-ethylmaleimide-sensitive factor (NSF); (c) cell proliferation, differentiation, and apoptosis anti-proliferative factor (BTG1), thyroid hormone receptor a1 (c-erb A α1); (d) metabolism such as stearoyl-CoA desaturase 2, beta isoform of catalytic subunit of cAMP-dependent protein kinase, ATP-citrate lyase. (e) RNA transcript and regulation such as Zinc finger gene, Jun-D gene, and ribosomal protein genes encoding larger ribosomal subunits L13, L8, and L22; (f) ion channel/pump such as potassium channel-Kv2, electrogenic Na+ bicarbonate cotransporter (NBC), type II Ca2+/calmodulin-dependent protein kinase beta subunit, and protein kinase C-regulated chloride channel.

**Table 3:** The results of RAF identifying genes differentially expressed among HS-SHRSPs, LS-SHRSPs, HS-SHRSRs, and LS-SHRSRs.

Delta	Number of genes called significant	Number of false discoveries	Estimated FDR
0.01253	3543	1181	0.333
0.74013	1504	500	0.332
1.10516	1157	173	0.150
1.47167	944	117	0.124
1.84017	794	83	0.105
2.21118	668	59	0.088
2.58527	580	44	0.076
2.96301	515	34	0.066
3.34503	437	24	0.055
3.73199	392	19	0.048
4.12463	370	15	0.041
4.52373	338	12	0.036
4.93017	307	10	0.033
5.34493	269	7	0.026
5.7691	250	6	0.024
6.20391	229	5	0.022
6.65078	209	4	0.019
7.11135	194	3	0.015
7.58753	182	2	0.011
9.13461	145	1	0.007
11.6257	107	0	<0.007

HS, high salt; LS, low salt; SHRSP, stroke-prone SHR/A3 (Heid) rats; SHRSR, stroke-resistant SHR/N (CRiv) rats.

### Independent verification of array findings

Fornage et al (2003) used TagMan assay to measure the relative expressions of 7 genes encoding atrial natriuretic peptide (Anp), the neurotrophin receptor protein tyrosine kinase (TrkB, short), casein kinase 2 (Ck2), complexin 2 (Cplx2), stearoyl CoA desaturase 2 (Scd2), glycerol-3-phosophate acyltransterase (Gpan), and inositol 1,4,5-triphosphate receptor (Itpr1). They found these 7 genes had significantly differentially expressed between SHRSP and SHR strains with p < 0.05. Except that genes Anp and Gpan were out of our data, genes for TrkB (short), Cplx2, and Scd2 called significant differential expressions at FDR<0.7%, and for CK2 and Itpr1 at FDR = 0.7% were found among HS-SHRSP, LS-SHRSP, HS-SHR, and LS-SHR strains. Interestingly, Tropea et al [27] also found the genes encoding glutamate receptor (GluR-A) and GABA receptor had significant expression difference between two groups of mice treated by dark rearing and monocular deprivation.

## Discussion

To our knowledge, the ranking analysis of *F*-statistics for identifying differentially expressed genes among multiple groups (classes) has not been reported. There are two main difficulties to be overcome: (a) estimate of the null *F*-distribution and (b) estimate of FDR. In conventional statistical methods, permutation is very popular to generate empirical distributions as estimates of the null distributions. However, the permutation approach may not be suitable for microarray data [10-13] because in general microarray experiments have a small sample size due to cost, as a result, treatment effect residues that cannot be removed are amplified in permutation distribution and resulting estimated null distribution has a heavier tail compared to true null distribution [12]. This would results in two consequences: (a) the estimated null distribution is not stable, which, as seen in Table 3, leads to low conservativeness of estimate of FDR, and (b) low power. Our RAF method is successful because the $\bar{f}$-distribution obtained by applying the RS approach [14] does not contain treatment effects and hence is a desirable estimate of the null *F*-distribution, which is supported by the fact that the observed and simulated results agree well with those expected by theory. Therefore, the number (M) of splits is much smaller than that of permutations for estimate of the null F-distribution. Simulation results showed that 50 splits are enough to obtain a stable and smooth $\bar{f}$-distribution. In addition, since the $\bar{f}$-distribution is generated from all the genes detected on microarrays and does not contain treatment effect residences, impact of sample size on the $\bar{f}$-distribution is very weak. However, we also noted that the $\bar{f}$-distribution would underestimate the null *F*-distribution when sample sizes are smaller than 4. In this situation, Eq. (7) should be changed to $\sigma^{2}({\bar{e}}_{g}^{J})=\sum_{i=1}^{n}4{\left({\bar{e}}_{gi}^{J}-{\bar{e}}_{g}^{J}\right)}^{2}/(n-1)$.

FDR is often used to control the error rate in the BH procedure [7], the BL procedure [8], and in SAM [9]. In practice, for a ranking test, it is necessary to obtain an accurate estimate of FDR. In SAM, FDR is estimated through the permutation approach in which fluctuations around expectation occur among permutated samples. The fluctuations would be dependent on the data itself, i.e., sample size, treatment effect, and data noise. In addition, as indicated above, permutation fails to remove the treatment effects in the data permuted from the microarray data with a small sample size so that the fluctuations are not purely due to random errors. Thus, this approach may give a biased estimate of FDR for a given threshold. The RAF estimator is based on a two-simulation strategy and hence avoids these problems of the SAM estimator, that is, its accuracy is not affected by sample size, treatment effect, and noise. As a result, the number (B) of simulations is also relatively small. Our simulation study indicates that more than 40 simulated data sets (B ≥ 40) would produce stable estimates of FDR across all given thresholds.

Our current RAF method can be readily extended to other test statistics such as Brown-Forsythe test statistic [28], Welch test statistic [29], and Cochran test statistic [30] by replacing F-statistic with the respective statistics.

## Conclusion

We developed a new statistical method that is suitable for analyzing microarray data to identify differentially expressed genes among multiple groups, especially, when sample size is small.

## Authors' contributions

YDT participated in the method development, performed the statistical analysis and drafted the manuscript. MF participated in the design of the study, carried out the microarray experiment. HX conceived of the study, and participated in its design and coordination and helped to draft the manuscript. All authors read and approved the final manuscript.

## Supplementary Material

Additional File 1 Genes with significant expression changes among four groups. The data provided are the tables containing detailed information of the 107 genes with significant expression changes among the four groups with FDR < 0.7%. Click here for file

## References

[B1] DeRisi J, Penland L, Brown PO, Bittner ML, Meltzer PS, Ray M, Chen Y, Su YA, Trent JM (1996). Use of a cDNA microarray to analyse gene expression patterns in human cancer. Nature genetics.

[B2] DeRisi JL, Iyer VR, Brown PO (1997). Exploring the metabolic and genetic control of gene expression on a genomic scale. Science.

[B3] Kim YD, Sohn NW, Kang C, Soh Y (2002). DNA array reveals altered gene expression in response to focal cerebral ischemia. Brain research bulletin.

[B4] Kerr MK, Martin M, Churchill GA (2000). Analysis of variance for gene expression microarray data. J Comput Biol.

[B5] Holm S (1979). A simple sequentially rejective multiple test procedure. Scandinavian Journal of Statistics.

[B6] Hochberg Y (1988). A sharper Bonferroni procedure for multiple tests of significance. Biometrika.

[B7] Benjamini Y, Hochberg Y (1995). Controlling the false discovery rate: a practical and powerful approach to multiple testing. Journal of the Royal Statistical Society Series B Methodological.

[B8] Benjamini Y, Liu W (1999). A step-down multiple hypotheses tesing procedure that controls the false discovery rate under independence. Journal of Statistical Planning and Inference.

[B9] Tusher VG, Tibshirani R, Chu G (2001). Significance analysis of microarrays applied to the ionizing radiation response. Proceedings of the National Academy of Sciences of the United States of America.

[B10] Zhao Y, Pan W (2003). Modified nonparametric approaches to detecting differentially expressed genes in replicated microarray experiments. Bioinformatics (Oxford, England).

[B11] Pan W (2003). On the use of permutation in and the performance of a class of nonparametric methods to detect differential gene expression. Bioinformatics (Oxford, England).

[B12] Xie Y, Pan W, Khodursky AB (2005). A note on using permutation-based false discovery rate estimates to compare different analysis methods for microarray data. Bioinformatics.

[B13] Gao X (2006). Construction of null statistics in permutation-based multiple testing for multi-factorial microarray experiments. Bioinformatics (Oxford, England).

[B14] Tan YD, Fornage M, Fu YX (2006). Ranking analysis of microarray data: a powerful method for identifying differentially expressed genes. Genomics.

[B15] Cui X, Churchill GA (2003). Statistical tests for differential expression in cDNA microarray experiments. Genome biology.

[B16] Li H, Wood CL, Liu Y, Getchell TV, Getchell ML, Stromberg AJ (2006). Identification of gene expression patterns using planned linear contrasts. BMC Bioinformatics.

[B17] Chen D, Liu Z, Ma X, Hua D (2005). Selecting genes by test statistics. Journal of biomedicine & biotechnology.

[B18] Tsai PW, Lee ML (2005). Split-plot microarray experiments: issues of design, power and sample size. Applied bioinformatics.

[B19] Cui X, Hwang JT, Qiu J, Blades NJ, Churchill GA (2005). Improved statistical tests for differential gene expression by shrinking variance components estimates. Biostatistics (Oxford, England).

[B20] Fornage M, Swank MW, Boerwinkle E, Doris PA (2003). Gene expression profiling and functional proteomic analysis reveal perturbed kinase-mediated signaling in genetic stroke susceptibility. Physiological genomics.

[B21] Lockhart DJ, Dong H, Byrne MC, Follettie MT, Gallo MV, Chee MS, Mittmann M, Wang C, Kobayashi M, Horton H (1996). Expression monitoring by hybridization to high-density oligonucleotide arrays. Nature biotechnology.

[B22] Benjamini Y, Yekutieli D (2001). The control of the false discovery rate in multiple testing under dependency. Annals of Statistics.

[B23] Storey JD (2002). A direct approach to false discovery rates. Journal of the Royal Statistical Society Series B Methodological.

[B24] Storey JD, Taylor JE, Siegmund D (2004). Strong control, conservative point estimation and simultaneous conservative consistency of false discovery rates: a unified approach. Journal of the Royal Statistical Society Series B Methodological.

[B25] Pounds S, Cheng C (2006). Robust estimation of the false discovery rate. Bioinformatics (Oxford, England).

[B26] Cho H, Lee JK (2004). Bayesian hierarchical error model for analysis of gene expression data. Bioinformatics (Oxford, England).

[B27] Tropea D, Kreiman G, Lyckman A, Mukherjee S, Yu H, Horng S, Sur M (2006). Gene expression changes and molecular pathways mediating activity-dependent plasticity in visual cortex. Nature neuroscience.

[B28] Brown MB, Forsythe AB (1974). The small sample behavior of some statistics which test the equality of several means. Technometrics.

[B29] Welch BL (1951). On the comparison of several mean values: An alternative approach. Biometrika.

[B30] Cochran WG (1937). Problems arising in the analysis of a series of similar experiments. Journal of Royal Statistics Society Serial C Applied Statistics.

