# Supplementary material for: Ranking analysis of F-statistics for microarray data
Source: BMC Bioinformatics. 2008 Mar 6;9:142. doi: 10.1186/1471-2105-9-142 (PMC2323973; doi:10.1186/1471-2105-9-142)
Supplement: Additional File 1 — Genes with significant expression changes among four groups. The data provided are the tables containing detailed information of the 107 genes with significant expression changes among the four groups with FDR < 0.7%. [file 1471-2105-9-142-S1.pdf]

Supplement table Genes with significant expression changes among four groups

Accession	F-value	Gene	Accession	F-value	Gene
X02002	135.805	cell-surface glycoprotein	U35099	27.073	**complexin II**
D64061	115.675	annexin V-binding protein (ABP-7)	D28111	26.421	MOBP (myelin-associated oligodendrocytic basic protein)
U41164	91.472	**Cys2/His2 zinc finger protein (rKr1)**	X52772cds	25.894	P65
rc_AI230256	85.863	EST226951 Rattus norvegicus cDNA,	rc_AA874934	25.841	UI-R-E0-ci-c-05-0-UI.s1 Rattus norvegicus cDNA
rc_AI638989	73.432	rc_AI638989	M55291	25.577	**receptor protein-tyrosine kinase (trkB)**
D26307cds	72.673	jun-D gene	X62145cds	25.291	ribosomal protein L8
X60212_i	64.678	mammalian equivalent of bacterial large ribosomal subunit protein L22	AF023621_at	25.078	sortilin
rc_AA859837	62.971	UI-R-E0-cc-g-09-0-UI.s1 Rattus norvegicus cDNA	AB012234_g_at	24.47	NF1-X1
rc_AI013194	58.552	EST207869 Rattus norvegicus cDNA	L14851_at	24.284	neurexin III-alpha
rc_AA875506	57.965	UI-R-E0-ct-c-05-0-UI.s1 Rattus norvegicus cDNA	rc_AI237576	23.904	EST234138 Rattus norvegicus cDNA
D28111	57.12	D28111	rc_H31692	23.7	EST106007 Rattus norvegicus cDNA
rc_AA875659	55.237	UI-R-E0-ct-h-07-0-UI.s1 Rattus norvegicus cDNA	D84477	23.587	RhoA
AF036761	49.617	stearoyl-CoA desaturase 2	X02610	23.473	non-neuronal enolase (NNE) (alpha-alpha enolase, 2-phospho-D-glycerate hydrolase EC 4.2.1.11)
X12744	47.901	c-erb-A thyroid hormone receptor	U73458	23.282	protein tyrosine phosphatase (PTPNE6)
E13644cds	44.43	Neurodap-1	D10770	23.119	beta isoform of catalytic subunit of cAMP-dependent protein kinase
AB000362	40.85	CIRP	S83025	22.98	TSH receptor suppressor element-binding protein-1
rc_AA893065	38.352	EST196868 Rattus norvegicus cDNA	J00692	22.736	skeletal muscle alpha-actin gene
AF001898	38.334	aldehyde dehydrogenase (ALDH)	AB016160	22.718	AB016160
S59158	35.44	glutamate transporter	L12382	22.672	ADP-ribosylation factor 3
U38653	35.344	olfactory inositol 1,4,5-trisphosphate receptor (InsP3R)	M27159cds	22.489	potassium channel-Kv2
rc_AI231213	33.851	EST227901 Rattus norvegicus cDNA	AB015432	22.324	LAT1 (L-type amino acid transporter 1)
X73653	33.317	tau protein kinase I	M92076	22.205	metabotropic glutamate receptor 3
AB016160	33.304	GABAB receptor 1c	M74494	22.191	sodium/potassium ATPase alpha-1 subunit truncated isoform
rc_AA924925	33.299	UI-R-A1-eg-d-06-0-UI.s1 Rattus norvegicus cDNA	L10326	22.047	alternatively spliced GTP-binding protein alpha subunit (stimulatory) (GS-alpha)
M36419	32.992	glutamate receptor (GluR-B)	X74402	21.966	GDI alpha
AF036761	32.527	**stearoyl-CoA desaturase 2**	U35774	21.869	cytosolic branch chain aminotransferase
rc_AA858621	32.32	UI-R-E0-bq-b-10-0-UI.s1 Rattus norvegicus cDNA	U14398	21.625	synaptotagmin IV homolog
L10362	31.279	synaptic vesicle protein 2B (SV2B)	M95735	21.429	syntaxin B
AF074482	31.083	GABA-B receptor 2 (GABA-BR2)	AF089839	21.404	N-ethylmaleimide sensitive factor
rc_AA858621	29.627	UI-R-E0-bq-b-10-0-UI.s1 Rattus norvegicus cDNA	AF083330	21.228	kinesin-like protein KIF3C (KIF3C)
rc_AA891069	29.623	EST194872 Rattus norvegicus cDNA	rc_AA859990	20.85	UI-R-E0-ca-a-08-0-UI.s1 Rattus norvegicus cDNA
X53565	29.286	trans-Golgi network integral membrane protein (TGN38)	L27075	20.701	ATP-citrate lyase
S75730	28.937	stearoyl-CoA desaturase 2 SCD2 homolog	rc_AI639314	20.603	rc_AI639314
rc_AA799406	28.111	EST188903 Rattus norvegicus cDNA	AB006451	20.527	Tim23

Continue Supplement table

Accession	F-value	Gene	Accession	F-value	Gene
AF022083	20.433	guanine nucleotide binding protein beta 1 subunit	U42627	17.451	dual-specificity protein tyrosine phosphatase (rVH6)
S45812	20.196	monoamine oxidase A	D14425	17.438	calcineurin B
M36418	20.049	glutamate receptor (GluR-A)	D17521	17.399	protein kinase C-regulated chloride channel
S81917	20.029	cysteine string protein=34 kDa DnaJ-hsp40 heat shock-chaperone protein	AF004017	17.353	electrogenic Na+ bicarbonate cotransporter (NBC)
M16112	19.849	type II Ca2+/calmodulin-dependent protein kinase beta subunit	U15408	17.35	plasma membrane Ca2+-ATPase isoform 4
U45479	19.665	synaptojanin	AF037071	17.321	carboxyl-terminal PDZ ligand of neuronal nitric oxide synthase
X12535cds	19.449	ras-related protein p23	rc_AA957961	17.268	UI-R-E1-fz-g-08-0-UI.s1 Rattus norvegicus cDNA
AFFX_ _M	18.974	Hexok	rc_AI070521	17.114	UI-R-Y0-lv-f-09-0-UI.s1 Rattus norvegicus cDNA
rc_AI104035	18.797	EST213324 Rattus norvegicus cDNA	rc_AA891729	17.074	EST195532 Rattus norvegicus cDNA
rc_AA894089	18.64	EST197892 Rattus norvegicus cDNA	X78327	16.979	ribosomal protein L13
rc_AA799538	18.613	EST189035 Rattus norvegicus cDNA	U08976	16.881	Wistar peroxisomal enoyl hydratase-like protein (PXEL)
rc_AA892378	18.58	EST196181 Rattus norvegicus cDNA	rc_AA859520	16.819	UI-R-E0-br-b-02-0-UI.s1 Rattus norvegicus cDNA
AF058795	18.574	GABA-B receptor gb2	X76724	16.806	RCK beta2
rc_AA893172	18.489	EST196975 Rattus norvegicus cDNA	U94189	16.689	Duo
rc_AI172162	18.145	EST218157 Rattus norvegicus cDNA	U39320	16.589	cysteine string protein
rc_AI172162	18.145	EST218157 Rattus norvegicus cDNA	D10587	16.461	85kDa sialoglycoprotein (LGP85)
AF055065	18.085	signal regulatory protein alpha	rc_AI012805	16.458	EST207256 Rattus norvegicus cDNA
L26268	17.814	anti-proliferative factor (BTG1)	rc_AI044508	16.372	UI-R-C1-kc-a-07-0-UI.s1 Rattus norvegicus cDNA
U62897	17.762	carboxypeptidase D precursor (Cpd)	rc_AA892801	16.306	EST196604 Rattus norvegicus cDNA
AB012231	17.514	NF1-B2			
